# Emotional Reactivity to Incentive Downshift in Adult Rats Exposed to Binge-Like Ethanol Exposure During Adolescence

**DOI:** 10.3389/fpsyg.2019.00315

**Published:** 2019-02-19

**Authors:** José Manuel Lerma-Cabrera, Camilo Andrés Arévalo-Romero, Gustavo Alfredo Cortés-Toledo, Alfredo Alfonso Adriasola-Carrasco, Francisca Carvajal

**Affiliations:** ^1^Centro de Investigación Biomédica, Facultad de Ciencias de la Salud, Universidad Autónoma de Chile, Santiago, Chile; ^2^Facultad de Psicología, Universidad de Oviedo, Oviedo, Spain; ^3^Facultad de Psicología, Universidad de Almería, Almería, Spain

**Keywords:** binge-like ethanol exposure, adolescence, intermittent-access ethanol paradigm, emotional state, successive negative contrast

## Abstract

Alcohol use in adolescents is often characterized by binge-like ethanol consumption pattern, which is associated with long-term health consequences and even with important harms to his developing brain. Among this, ethanol exposure induces long-lasting alterations in anxiety-related neurobiological systems such as corticotropin releasing factor (CRF) or melanocortin system (MC). Recently, it has been demonstrated that adult rats exposed to adolescent intermittent ethanol (AIE) exposure exhibited anxiogenic-like behavior. Given that it has been demonstrated that negative affective state is relevant to development of addictive behavior, it is tempting to suggest that increased risk of adult abusive alcohol use exhibited in rats exposed to ethanol during adolescence may be related with differences in anxiety-related behavior. We conducted a study investigating the emotional reactivity after a reward devaluation (12-to-1 pellet or 32-to-4% sucrose downshift) in adult rats exposed to binge-like ethanol exposure during adolescence. For this aim, adolescent Sprague-Dawley rats were treated with ethanol (2.5 g/kg ip; AIE) or saline (AIS) for 2 consecutive days at 48-h intervals over a 14-day period (PND30-PND43). Following 25 free-ethanol days, adult rats were trained in consummatory and instrumental successive negative contrast task (cSNC and iSNC). Our data shows that both AIE and AIS groups exhibited suppression of the consummatory and instrumental behavior after reward devaluation relative to unshifthed control. Also, adult rats exposed to alcohol during adolescence exhibited a particularly strong negative affective state (lower sucrose consumption) with regards to the AIS group in the cSNC. This data demonstrated that adolescent binge-like ethanol exposure might trigger a greater emotional reactivity following incentive downshift, which might be linked to higher vulnerability to substance use disorder.

## Introduction

According to National Institute on Alcohol Abuse Alcoholism [NIAAA] (2017), almost 90% of alcohol consumed by adolescents is in the form of binge-drinking, especially during leisure time and weekends; namely, drinking 4/5 standard alcohol drinks (women/men, respectively) in a 2 h timeframe, that usually results in high blood alcohol concentration (about 0.8 mg/dl). The prevalence of binge drinking among adolescents (15–19 years) in Europe and America is 24.1 and 18.5% respectively (World Health Organization [WHO], 2018). However, in some countries of South America such as Argentina, Chile or Peru this pattern of ethanol consumption is particularly prevalent with one out of every two students showing binge drinking in the last month (Organization of American States [OAS], 2013; Pillati et al., 2017). Even though alcohol use increases with age, and sometimes even the total amount of alcohol consumed per month is greater in adults than in adolescents, it has been estimated that adolescents consume larger amount of alcohol per occasion than adults (Carvajal and Lerma-Cabrera, 2015).

Underage binge-drinking is particularly dangerous because adolescence is an important period for brain development where ethanol exposure causes long-lasting neuroadaptative changes in neural pathways critically involved with neurobehavioral responses to ethanol (Pascual et al., 2007, 2009, 2012; Maldonado-Devincci et al., 2010; Lerma-Cabrera et al., 2013a). Several studies had associated early alcohol use with increased likelihood of abusive alcohol use and other anxiety-related disorders in adulthood (Pascual et al., 2009; Maldonado-Devincci et al., 2010; Moaddab et al., 2017). Importantly, adolescent intermittent ethanol exposure alters basal α-MSH, NPY and CRF activity in the amygdala and hypothalamic areas (Przybycien-Szymanska et al., 2011; Gilpin et al., 2012; Lerma-Cabrera et al., 2013a; Kokare et al., 2017). Given that these neurobiological systems are involved not only in the regulation of ethanol consumption but also in anxiety and emotional stress (Koob, 2013; Berkel and Pandey, 2017), several studies have suggested that dysregulation of emotional processing, similar to the observed in adolescent binge drinkers, may drive compulsivity in ethanol intake (Koob, 2013, 2015).

It has been demonstrated that binge alcohol-drinking elicits symptoms of negative affect such as ethanol withdrawal-induced anxiety in adult mice (Lee et al., 2015) or social anxiety (Varlinskaya et al., 2014). In many case, the emergence of a negative emotional state (e.g., dysphoria, anxiety and irritability) leads to seek the drug to remove it. It has been called the “dark side” of addiction and plays an important role in the maintenance of drug dependence (Koob, 2013, 2015). However, the adverse impact of binge-like ethanol exposure during adolescence on other aspects of negative emotional states like frustration has received relatively limited attention.

Successive negative contrast (SNC) is a reward downshift or incentive downshift procedures widely used to evaluate the emotional state in animal models (Flaherty, 1996). The discrepancy between the actual reward of low value and the expected reward of higher value causes a reduction in instrumental or consummatory behavior and elicits an aversive emotional, cognitive and behavioral state called frustration (Amsel, 1958, 1992). In this way, it has been found that exposure to reward devaluation activates the hypothalamic–pituitary–adrenal (HPA) axis and increases the release of stress hormones (i.e., ACTH and corticosterone) (Papini and Pellegrini, 2006; Pecoraro et al., 2009). Also, behavioral response induced by reward devaluation is reduced by anxiolytic drugs such as diazepam (Mustaca et al., 2000; Kamenetzky et al., 2008) or ethanol (Manzo et al., 2015).

Genetic and lesion studies also suggest the role of emotional behavior in reward devaluation situations. In this way, several studies have shown that rats who are selectively bred to exhibit differences in emotional reactivity, fearfulness or anxiety also differ in their response to reward loss. This is the case of Roman high-avoidance (RHA) vs. low-avoidance (RLA) rats (Rosas et al., 2007; Gómez et al., 2008; Cuenya et al., 2015), Lewis vs. Fischer rats (Brewer et al., 2016) or spontaneously hypertensive vs. Wistar-Kyoto rats (Bentosela and Mustaca, 2005) among others. Consistent with genetic studies, lesions in brain areas involved in emotional responses such as nucleus accumbens, the hippocampus, the medial prefrontal cortex or the amygdala disrupt the SNC effects (Becker et al., 1984; Flaherty et al., 1998; Leszczuk and Flaherty, 2000). Specifically, lesions of the hippocampus decrease the occurrence of instrumental SNC (iSNC), while local inactivation of the centromedial amygdala reduce (Bentosela et al., 2001; Kawasaki et al., 2015). The excitotoxic lesion of basolateral amygdala eliminate the consummatory SNC (cSNC) effect (Kawasaki et al., 2017). These studies suggest that the neural circuitry underlying both procedures (iSNC and cSNC) are different.

Recent studies suggests a bidirectional interaction between reward loss and addictive behavior (see review Ortega et al., 2017). In this way, it has been shown that the reward devaluation increases ethanol consumption and preference (Manzo et al., 2015). Besides, Matson and Grahame (2015) reported that High Alcohol Preferring (HAP) mice, which are selectively bred to prefer ethanol, exhibited larger cSNC effects compared to their corresponding replicate Low Alcohol Preferring (LAP) lines. Also, pharmacological studies have shown that drugs with addictive potential (e.g., opioids or dopaminergic drugs) have an impact on reward loss (Barr and Phillips, 2002; Daniel et al., 2009). Thus, it has been suggested that SNC could be a useful tool to better understanding the initiation and maintenance of alcohol use disorders (AUD).

Previous studies have reported that exposure to alcohol during adolescence resulted in alterations of neuropeptides involved in processing of negative emotional reactions (Koob, 2013; Berkel and Pandey, 2017). Given that it has been demonstrated that negative affective state is relevant to development of addictive behavior, it is tempting to suggest that an increased risk of adult abusive alcohol use exhibited in rats exposed to ethanol during adolescence (Pandey et al., 2015) may be related with differences on negative emotional states. To address this issue, the main objective of the present study was to evaluate whether binge-like ethanol exposure during adolescence alters response of frustration in adulthood through a task of SNC. Additionally, we evaluated whether binge-like ethanol exposure during adolescence differentially affect the iSNC and cSNC in adulthood.

## Materials and Methods

### Animals

Sixty-one male Sprague-Dawley rats on postnatal day (PND 30) (Pontificia Universidad Católica de Chile, Chile) were used as subjects in these experiments. The rats remained housed in groups of three/four rats per cage and maintained in an environmentally controlled room (22°C temperature in a 12:12 h light-dark cycle). Standard rodent chow and water were provided *ad libitum*. In adulthood (PND 60), the animals were housed individually and assigned randomly to reinforcement groups. Throughout the experiment, rats were deprived to 80–90% of their *ad lib* weight via daily feedings of lab chow, approximately 30 min after the end of the experimental session. Animals had free access to water throughout the experiment. Behavioral procedures were approved by the University of Autonoma of Chile Bioethical Animal Care and Use Committee.

### Apparatus

#### iSNC Task

The apparatus was a black straight Plexiglas runway measuring 120 × 11 × 14 cm (L × W × H), and covered by clear Plexiglas lids. The runway was divided into three sections. The start and goal box were 20cm long and were separated from the running section by two guillotine doors. As reward a 45-mg pellets (formula P; Research Diets, Lancaster, NH, United States) [either 1 or 12 pellet(s), depending on the group] were placed on the floor at the distal end of the goal box. Response latencies (in seconds) were measured with the help of a chronometer (Extech, model 364410) as time from the moment the guillotine door was raised to the moment the rat had all four its legs inside the goal box.

#### cSNC Task

Consummatory training was conducted in Plexiglas boxes (measuring 30 × 15 × 30 cm) with a central orifice through which animals had access to a bottle containing a 32% or 4% (w/v) sucrose solution during 5 min. After this period, sucrose consumption (g) was assessed.

### Procedure

#### Ethanol Exposure

Morning doses of either 25% (w/v) ethanol (2.5 g/kg) in isotonic saline (adolescent intermittent ethanol group, AIE), or saline (adolescent intermittent saline group, AIS) were intraperitoneally administered (i.p.) to 30-day old rats on two consecutive days with gaps of 2 days without injections, during 2 weeks. Specifically, rats were injected at PND 30, 31, 34, 35, 38, 39, 42, and 43 (Lerma-Cabrera et al., 2013a; Carvajal et al., 2017).

#### SNC Task

##### Experiment 1: incentive downshift through iSNC task

On PND 66, thirty-one rats (16 AIE and 16 AIS) were tested in the instrumental SNC following the prior described procedure (Flaherty et al., 1998; Rosas et al., 2007). Specifically, the experiment was conducted in three phases: habituation, preshift, and postshift phases.

###### Habituation

On the first day, rats were allowed to freely explore the entire runway for five trials of 2 min each. On the second day, rats were given two 2-min trials of free-access followed by three feeding in the goal box (i.e., the animal was confined to the goal box and given the appropriate number of pellets either 12 or 1 depending on the group). The last habituation session consisted of three goal-box feeding trials in which subjects have a maximum of 30 s to consume the pellets. After that, they were removed from the goal box and taken back to their home cage.

###### Preshift training phase

After this habituation, each animal was placed in the start box with the door closed and the goal door opened. Each session began with the opening of the start door and finished when the rat reach the goal box to obtain the food reward [1 or 12 pellet(s), depending on the group]. A maximum time of 20 s was allowed for the rat to complete the trial. If the rat did not reach the goal box before 20 s had elapsed, it was gently pushed into the goal box by the experimenter and 20 s was recorded as response latency. When the rat reached the goal box, the goal door was quietly closed and a stopwatch was stooped. The rat was given a maximum of 30 s to consume the food reward. As soon as the rat had finished eating or 30 s had elapsed, it was removed from the goal box and placed back in its home cage between trials.

###### Postshift phase

On day 10, the last trial of training, the rats receiving 12 pellets were shifted to 1 pellet while the rats receiving 1 pellet remained unchanged. Thus, from the 10th day of training onward, all rats received 1 pellet upon arrival to the goal-box. The postshift phase continued for 2 days.

##### Experiment 2: incentive downshift through cSNC task

Consummatory SNC task were based on previous procedures (Cuenya et al., 2015; Manzo et al., 2015). On PND 66, rats (*n* = 15 for AIE group and *n* = 14 for the AIS group) were familiarize with the consummatory box and allowed to freely explore the box for 5 min. On Days 2 and 3 of the habituation period, the animals also have access to a bottle containing water.

###### Preshift phase

On Days 4–13, half of the animals in each group (AIE or AIS) received free access to 32% sucrose solution and the remaining half of animals received 4% sucrose solution in the consummatory box. Immediately after the first contact with the sipper tube, sucrose intake (ml) was recorded for 5 min in each session.

###### Postshift phase

During the next two sessions (days 14–15), all animals were exposed to 4% sucrose for 5 min.

### Data Analysis

Kolmogorov–Smirnov–Lilliefors (K–S–L) test indicated that, across datasets, the assumptions of homogeneity and normality were maintained. The sphericity assumption was tested with W of Mauchly’s statistic. In case of not fulfilling the assumption, it was used as a corrector index Huynh-Feldt (H-F).

Repeated measures of ANOVA using Days as the repeated measure factor and Treatment (AIE vs. AIS) and Reinforcement (12-1 and 1-1 pellets or 32-4 and 4-4 percentage of sucrose) as between-subjects measures was used to analyze preshift data both in iSNC and cSNC. A similar repeated measures of ANOVA was used to analyze the effect of reward downshift, including the last preshift and postshift session. When significant differences were found (*p* < 0.05), pair wise comparisons were conducted with *post hoc* Newman–Keuls test. Partial eta-squared ($\eta_{p}^{2}$) was used as the measure of effect size for all analyses of variance (ANOVAs), with small ($\eta_{p}^{2}$ = 0.009), medium ($\eta_{p}^{2}$ = 0.09), and large ($\eta_{p}^{2}$ = 0.25) effect sizes (Levine and Hullett, 2002). All data in this report are presented as means ± SEM.

## Results

### Experiment 1: Incentive Downshift Through iSNC Task

**FIGURE 1:**
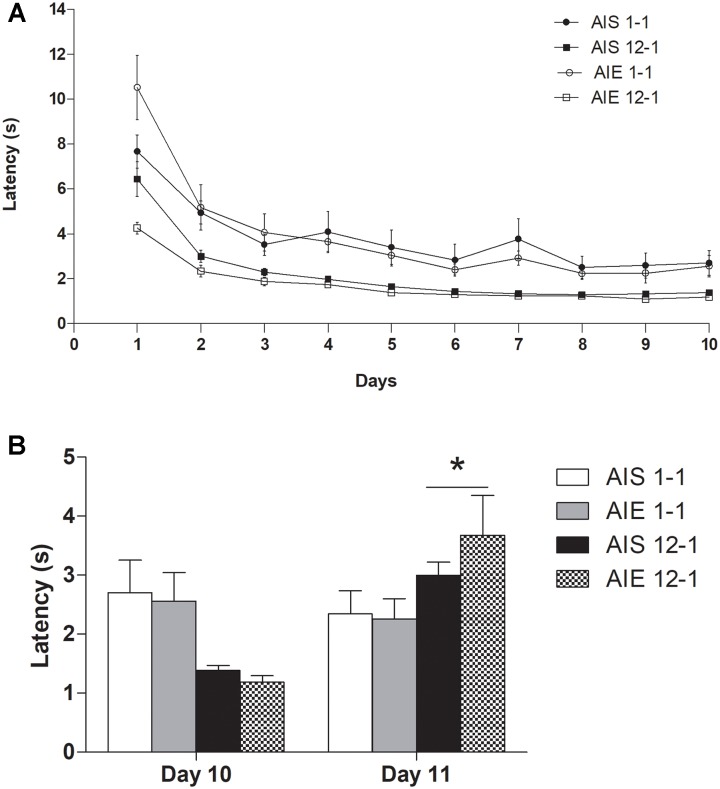
**(A)** Mean latency (s) to reach the goal-box on each of the training sessions in the straight alley in preshift phase and **(B)** in postshift phase in groups AIE12-1, AIE1-1, AIS12-1, and AIS1-1. All values are means ± SEM. ^∗^*p* < 0.05.

Figure 1A shows the mean latency (sec) to reach the goal-box during the preshift phase as function of receiving 12 or 1 pellet(s) for AIE and AIS group. Mauchly test indicated that the assumption of sphericity had been violated [χ2(44) = 199.233, *P* < 0.0001]; therefore, degrees of freedom were corrected using Huynh-Feldt estimate of sphericity. A Treatment × Reinforcement × Days analyses conducted for the first ten sessions (preshift phase) showed that rats reinforced with 12 pellets exhibited lower latency than those exposed to only 1 pellet, as shown by significant effects for Reinforcement [*F*(1,28) = 24.99, *p* < 0.0005; $\eta_{p}^{2}$ = 0.47] and for the Reinforcement × Days interaction [*F*(3.968,111.11) = 4.54, *p* < 0.00001; $\eta_{p}^{2}$ = 0.14]. There was also a Days effect [*F*(3.968,111.11) = 78.98, *p* < 0.00001; $\eta_{p}^{2}$ = 0.73] and Treatment × Reinforcement × Days interaction [*F*(3.968,111.11) = 5.10, *p* < 0.00001; $\eta_{p}^{2}$ = 0.15]. *Post hoc* analysis aimed to further analyze the triple interaction indicated greater latency in the AIS 1-1 group than the rest of the groups during day 1 (*p* < 0.05).

When we compared the latency to reach the final box during days 10 and 11 (when the downshift occurred), there was a significant effect for the Reinforcement × Days interaction [*F*(1,28) = 24.007, *p* < 0.00005; $\eta_{p}^{2}$ = 0.461]. *Post hoc* test revealed that rats that had been trained to run for 12 pellets showed higher latency in the post shift phase (day 11) than the rats that had been trained to run for 1 pellet from the outset (*p* = 0.03). Given that no other main effect or interaction was significant, this data suggests that binge-like ethanol exposure in adolescence did not alter differently the emotional response induced by the downshift in reward in iSNC task during adulthood. Figure 1B shows the average latency of each group in the last day of preshift (day 10) and postshift phases of iSNC.

### Experiment 2: Incentive Downshift Through cSNC Task

**FIGURE 2:**
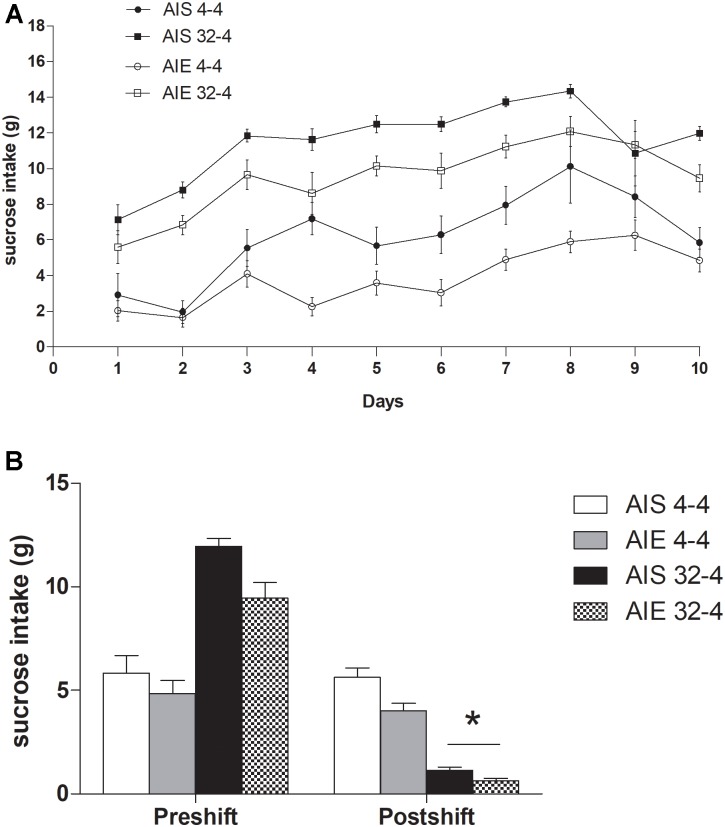
**(A)** Mean values of sucrose consumption (g) on each of the training sessions in preshift phase and **(B)** in postshift phase in both groups AIE32-4, AIE4-4, AIS32-4, and AIS4-4. All values are means ± SEM. ^∗^*p* < 0.0001.

Figure 2A shows the mean sucrose intake (g) in the four groups (AIE 32-4%, AIE 4-4%, AIS 32-4%, and AIS 4-4%) during the preshift phase. A 2 (Treatment) × 2 (Reinforcement) × 10 (Days) ANOVA conducted in the preshift phase revealed a significant effect of Treatment [*F*(1,25) = 14.727, *p* ≤ 0.001; $\eta_{p}^{2}$ = 0.37], Reinforcement [*F*(1,25) = 92.198, *p* ≤ 0.00001; $\eta_{p}^{2}$ = 0.79] and Days [*F*(3.968,202.128) = 31.567, *p* ≤ 0.00001; $\eta_{p}^{2}$ = 0.56]. Also, Treatment × Days [*F*(3.968,202.128) = 2.2266, *p* ≤ 0.02; $\eta_{p}^{2}$ = 0.08], and Reinforcement × Days interaction [*F*(3.968,202.128) = 2.1288, *p* ≤ 0.02; $\eta_{p}^{2}$ = 0.07] were statistically significant. However, the Treatment × Reinforcement and the triple interaction were not. The *post hoc* analysis of the Treatment × Days interaction revealed that animals pre-exposed to binge-like ethanol exposure during adolescence exhibited lower sucrose intake than AIS group from day 4 to day 8 of the preshift phase. In addition, the effect of the sessions was significant in both AIE [*F*(9,126) = 21.876, *p* ≤ 0.001; $\eta_{p}^{2}$ = 0.61] and AIS groups [*F*(9,117) = 13.373, *p* ≤ 0.01; $\eta_{p}^{2}$ = 0.51] showing an improvement in performance of the task across session. Additional *post hoc* tests aimed to further analyze Reinforcement × Days interaction revealed that animals receiving the 32% sucrose solution performed better than animals exposed to 4% sucrose throughout the training.

Data showing sucrose consumption (g) during the last preshift session and the postshift session (day 11) are represented in Figure 2B. The ANOVA performed on reward devaluation found a significant main effect of Treatment [*F*(1,25) = 10.226, *p* ≤ 0.004; $\eta_{p}^{2}$ = 0.29] and Days [*F*(1,25) = 306.09, *p* ≤ 0.00001; $\eta_{p}^{2}$ = 0.924]. The interaction Reinforcement × Days [*F*(1,25) = 247.54, *p* ≤ 0.00001; $\eta_{p}^{2}$ = 0.908] and Treatment × Reinforcement × Days [*F*(1,25) = 4.8447, *p* ≤ 0.03; $\eta_{p}^{2}$ = 0.162] were statistically significant. As was observed in training, AIE group drink less sucrose than AIS group also in the postshift phase (*p* = 0.004). Finally, on day 11 downshifted groups (AIE 32-4 and AIS 32-4) performed significantly below their respective unshifted control (AIE 4-4 and AIS 4-4) (*p* = 0.0001 in both AIE and AIS group).

Taking together, this data suggests that although binge-like ethanol exposure during adolescence did not alter the response of incentive downshift through cSNC task during adulthood, it reduced sucrose intake in both AIE 32-4 and AIE 4-4%.

## Discussion

Binge drinking, which is the most common pattern of ethanol consumption during adolescence, is a significant risk factor for the development of alcoholism. It is known that aversive emotional states may drive escalated ethanol intake in rodents, suggesting that increased likelihood of abusive alcohol use in rats exposed to ethanol during adolescence may be related with differences in anxiety behavior. This study assessed for the first time the effect of adolescence binge-like ethanol exposure on the response of frustration following a situation of reward devaluation in adulthood. To this end, AIE and AIS groups received training in the instrumental (iSNC) and consummatory successive negative contrast (cSNC) procedure. The results indicated that both AIE and AIS rats showed similar emotional reactivity to incentive downshift in instrumental and consummatory task. Thus, our results replicate previous studies on successive negative contrast following reward downshift and provide more evidence about the fact that negative successive contrast can be produced using reinforcers from different modalities (i.e., liquid vs. solid food). Also, AIE exhibited a generalized decrease of sucrose consumption in cSNC when compared with AIS group, suggesting that exposure to alcohol in adolescence might increase anhedonic behavior or palatability changes in adulthood.

In experiment 1, the results obtained during the preshift phase showed that all groups learned the task during the days shorter latencies were exhibited. Consistent with previous studies (Leszczuk and Flaherty, 2000; Rosas et al., 2007; Binkley et al., 2014), rats receiving 12 pellets showed lower latency than rats receiving 1 pellet. Binge-like ethanol exposure during adolescence had not an effect on latency during the preshift phase, with the exception of day 1 in which AIE/1-1 exhibited the major latency to reach the goal-box, in comparison with the rest of the groups. Several studies had shown that ethanol exposure during adolescence reduce spontaneous locomotor activity (Pascual et al., 2009; Teixeira et al., 2014; Fernandez and Savage, 2017). Thus, the increase in latency showed by group AIE/1-1 could be due to a low exploratory behavior in a novel context. However, as on this first day AIE/12-1 group exhibited lower latency, rather than higher, this explanation is unlikely. Conversely, it is tempting to postulate that in day 1, different reinforcing values of 1 pellet vs. 12 pellets in AIE group may have been a factor in the time spent to reach the goal-box on day 1. However, those differences are only observed on the first day and, given that exclusive measures of latency were taken, it is difficult to defend this idea. Additional measures as operant behavioral procedure among others are needed to specifically evaluate this hypothesis.

Some interesting data obtained on cSNC task also point to possible basal differences in the AIE group in the response to the reinforcing properties of reward. Although both groups (AIE and AIS) showed higher sucrose intake of the greater concentration of solution, animals exposed to binge-like ethanol during adolescence drinks significantly less sucrose compared to AIS group. This effect was independent of the magnitude of the presented reinforcer. Interestingly, these differences had been observed not only during the preshift phase but also during postshift phase. Generally, reduced sucrose intake is considered an index of anhedonia in animal models (Katz, 1982). Recently, it has been proposed that anhedonic behavior might arise from dysfunctional interaction between the stress and reward system (Pizzagalli, 2014; Bolton et al., 2018).

In this sense, exposure to alcohol during adolescence increases adult CRF mRNA expression in the hypothalamic paraventricular nucleus, central nucleus of the amygdala and the prefrontal cortex (Przybycien-Szymanska et al., 2011; Boutros et al., 2018, 2016). Such alterations are important due to the implication of CRF in anxiety (Binder and Nemeroff, 2010) and stress response (Deussing and Chen, 2018). Adolescent alcohol exposure also alters other anxiety-related neuropeptides such as α-MSH or NPY (Lerma-Cabrera et al., 2013a; Kokare et al., 2017). An increase in anxiety levels might interfere with the ability to experience pleasure during normally rewarding activities, such as the consumption of sucrose (Bolton et al., 2018). In relation with this idea, for example, it has been demonstrated that inbred RLA rats, which were selectively bred to display enhanced anxiety/fearfulness, exhibits elevated CRF gene expression in the extended amygdala in comparison with RHA rats (Carrasco et al., 2008). Besides, RLA 32-4% rats consume less sucrose than RHA 32-4% rats in the preshift phase of cSNC task (Gómez et al., 2008). Thus, our data of the reduction of sucrose intake in AIE groups could reflect an anhedonic response mediated by CRF or even by α-MSH given that stress-elicited anhedonia requires α-MSH/MC4R signaling (Lim et al., 2012). In addition, given that α-MSH has the ability to modulate non-homeostatic aspect of a reward (Lerma-Cabrera et al., 2013b), it should be considered that reduction of sucrose intake might be determined by changes in taste palatability or hedonic value of sucrose. Future studies analyzing the temporal microstructure of the consumption response (Dwyer, 2012) or using the taste reactivity test (Cuenya et al., 2018) are needed to evaluate the hedonic value of sucrose in AIE and AIS group. Otherwise, additional pharmacological or genetic studies directed to manipulate CRF or MC system will provide information for a deeper understanding of the involvement of these systems on anhedonia.

In the last decade, some studies had shown that neural circuit engaged in consummatory and instrumental SCN are, almost partially, different. Lesion studies suggests hippocampus and nucleus accumbens affect behavior in the iSNC situation (Leszczuk and Flaherty, 2000), whereas cSNC was affected by lesion of the amygdala and cortical areas (Bentosela et al., 2001; Ortega et al., 2013; Kawasaki et al., 2017). This neuroanatomical dissociation between iSNC and cSNC suggests that these tasks could involve relatively different emotional and cognitive mechanisms (Papini and Pellegrini, 2006). The amygdala has an important role not only in anxiety responses but also in reward learning and addiction (Janak and Tye, 2015; Kawasaki et al., 2017); for that, and as showed in our study, cSNC seems to be more sensitive than iSNC to evaluate changes in incentive salience after adolescent intermittent ethanol exposure.

The present study had shown a successive negative contrast effect in two testing situations (consummatory behavior and instrumental learning) in both AIE and AIS groups. In the potshift phase of iSNC, rats expecting a high-value reward (12 pellets) exhibited greater latency when given a low-value reward (1 pellet) relative to unshifted rats always receiving 1 pellet. It has been demonstrated that iSNC involving the devaluation from 12 to 1 pellet induce a negative emotional state due to incentive loss (Manzo et al., 2012; Papini et al., 2015). Secondly, and in accordance with other studies (Gómez et al., 2008; Manzo et al., 2015) a response of reduced intake of sucrose was observed when the concentration was severely reduced (from 32 to 4%) in the postshift phase of the cSNC. These results showed that the effect of deterioration in the performance of the shifted group was not related with previous history of alcohol intermittent exposure during adolescence. Several studies had shown that adolescent binge-drinking has long-lasting effects on anxiety-like behavior (Varlinskaya et al., 2014; Lee et al., 2015), however, in our study, at least under our experimental conditions, AIE and AIS animals did not exhibit differences in their responses when the amount of reinforcement was unexpectedly reduced in a consummatory or instrumental appetitive learning task. Other studies have also found controversial results: decreased anxiety or increased impulsivity (Gilpin et al., 2012) or even, no signs of anxiety (Marco et al., 2017) after adolescent intermittent alcohol exposure. The contrasting results in these studies may be related to the use of different protocol of ethanol exposure during adolescence, as well as the paradigm used to evaluate anxiety-related responses. Further studies are needed to clarify the effect of binge-like ethanol exposure on anxiety in adulthood. One possible explanation of our data is that a negative contrast effect is too powerful to be sensitive to distinguish between the performance of AIE and AIS group. Maybe, the reward downshift was aversive enough to cover an effect of adolescent intermittent ethanol exposure on the magnitude of SNC. Therefore, a ceiling effect cannot be ruled out. Bearing this in mind, reducing the reward disparity between the pre- and postshift phase (i.e., 12-2 in the iSNC and 22-4% in the cSNC) could increase the sensitivity of the tasks as suggested in Rosas et al. (2007) and Gómez et al. (2008). Also, identifying profiles of recovery from successive negative contrasts could help us test in major detail the effect of binge-like ethanol exposure during adolescence on negative emotional state has. It has been demonstrated that the higher level of suppression after the devaluation shown by animals, the lower level of recovery (Bentosela and Mustaca, 2005; Rosas et al., 2007; Gómez et al., 2008; Brewer et al., 2016).

In summary, the present study demonstrates that negative contrast in consummatory and instrumental behavior occurred after reward devaluation, regardless of binge-like ethanol exposure during adolescence. However, as showed in cSNC, animals exposed to binge-like ethanol during adolescence exhibited reduced sucrose intake. Besides, cSNC may be more sensitive to evaluate emotional changes induced by adolescent intermittent ethanol exposure than iSNC. The primary limitation to the generalization of these results could be the small group size (*n* = 8-7). But also, it is possible that small group size prevented us from findings more significant differences. Still, the small population did not negate recognition of the importance of binge drinking during adolescence on response of frustration in adulthood. Many similar studies evaluating emotional reactivity to incentive downshift in the instrumental and consummatory devaluation have reported a similar group size (Rosas et al., 2007; Gómez et al., 2008) or even a smaller number of animals (Cuenya et al., 2015). Future studies characterizing the motivational aspects and emotional state associated with reward loss could contribute to further understanding in which conditions intermittent ethanol exposure during adolescence leads to substance use disorder in adulthood.

## Author Contributions

JL-C and FC were responsible for the study concept and design. CA-R, GC-T, and AA-C conducted the experiments. FC, JL-C, CA-R, and GC-T analyzed the data. All the authors critically reviewed content and approved final version for publication.

## Conflict of Interest Statement

The authors declare that the research was conducted in the absence of any commercial or financial relationships that could be construed as a potential conflict of interest.
